# Rock mineral wool–based green roofs to improve the quality of urban water runoff

**DOI:** 10.1007/s11356-025-36232-7

**Published:** 2025-03-18

**Authors:** Gabriel Pérez, Julià Coma, Cristina Chocarro, Alejandro Juárez, Claudia Marín, Franc Rauter, Neva Zupanc, Barbara Šubic, Darja Majkovič

**Affiliations:** 1 Innovative Technologies for Sustainability (It4s) Research Group, University of Lleida, C/Jaume II 69, 25001 Lleida, Spain; 2 Department of Agricultural and Forest Sciences and Engineering, University of Lleida, C/Rovira Roure 191, 25198 Lleida, Spain; 3 Knauf Insulation d.O.O., Trata 32, 4220 Škofja Loka, Slovenia; 4 Municipality Zalec, Ul. Savinjske Cete 5, Zalec, Slovenia

**Keywords:** Nature-based solutions, Water quality, Pollution, Leachate, Green roof, Rock mineral wool

## Abstract

Green roofs are nature-based solutions that allow greenery to be integrated into the building envelope, making it possible to re-nature cities while providing multiple benefits. However, whether green roofs are a source or sink of pollution in the urban environment is still a controversy. One of the causes of the possible deterioration of the quality of runoff water from green roofs is the substrate. Green roofs based on rock mineral wool (RMW) growing media require thinner substrate layers or can even be substrate-less. In the present study, four green roof systems based on RMW have been studied over the course of 2 years. Their performance, in terms of leachate quality, has been compared with two traditional roofs, a green roof with pozzolana as a draining material and a gravel-ballasted conventional flat roof. Limit values for wastewater quality from international regulations were considered benchmark. The main conclusions were that after the first flush, which was observed for all solutions, generally exceeding the limit values, RMW-based solutions performed better than traditional solutions. Furthermore, the average values of leachates from all tested green roofs and especially those from RMW solutions fall within the limits set by international regulations.

## Introduction

In recent years, the world has become more than half urban for the first time in history, 57% in 2022 (Demographia 19th 2023). The urban lifestyle not only involves a disconnection from nature but also implies a set of environmental issues arising from urbanization processes and cities’ metabolism, including soil sealing and urban runoff, air and water pollution, loss of biodiversity, and the urban heat island (UHI) effect. Nature-based solutions (NBSs) have been consolidated in recent years as one of the most important strategies on the road to building future resilient, sustainable, and circular cities within the UN Sustainable Development Goals (SDG11. Sustainable cities and communities) (Maes et al. 2021; Marchal et al. 2019). In previous research, the transversal nature of NBS, specifically green roofs, in improving the sustainability of the built environment was highlighted. Results indicate that interventions motivated primarily by water management goals, such as green roofs, can considerably reduce energy use and contribute to mitigation of greenhouse gas emissions (Ericsdotter et al. 2017). The EU Research and Innovation Policy Agenda on Nature-based Solutions and Re-naturing Cities defines nature-based solutions to societal challenges as “solutions that are inspired and supported by nature, which are cost-effective, simultaneously providing environmental, social and economic benefits and help build resilience” (Adina and Wendling 2021).

**Fig. 1:**
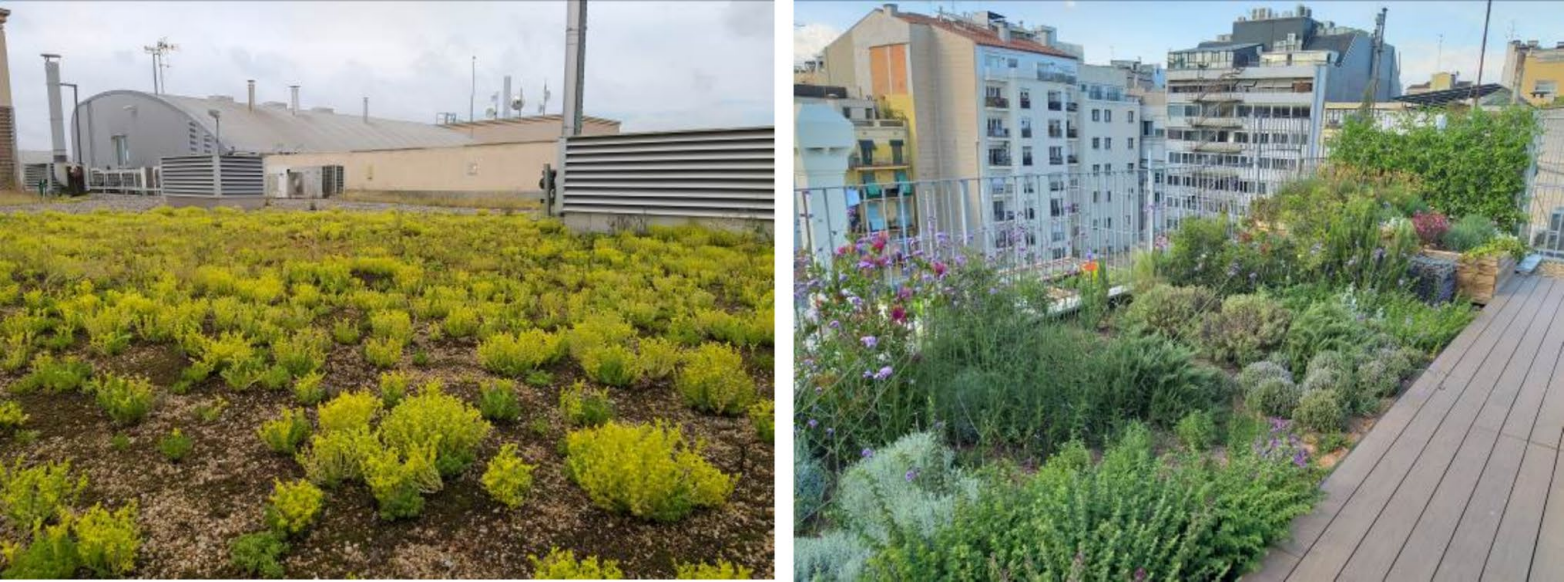
Left: extensive green roof in Lleida, Spain. Right: semi-intensive green roof in Barcelona, Spain

Among the multiple NBSs applicable on the city level, those that make it possible to integrate vegetation and nature into building envelopes, such as green roofs, green walls, and green facades, stand out for their ability to re-fill those scarce “opportunity” surfaces in the densely built environment. These systems facilitate the re-naturing of cities when there is no possibility to consider the traditional solutions, e.g., urban parks, gardens, street trees, or urban forests, among others (Fig. 1). The most widely used building-integrated systems are green roofs, in all their variants (extensive, semi-intensive, and intensive) depending on their level of construction complexity, level of maintenance, and capacity to support more developed vegetation (FLL guidelines 2018).

In the past, extensive research has been carried out on the benefits and criticalities of green roofs, the former being much more numerous than the latter, in a context of sustainable construction. The provision of benefits from green roofs takes place both at the building and city level. At the building level, green roofs improve building performance, providing energy savings and acoustic insulation. In addition, they improve and allow open spaces to be recovered within the urban environment, where landscaping, food production, and social activities can therefore be developed (Berardi et al. 2014; Todorov et al. 2018). At the urban level, they mitigate the urban heat island effect, contribute to storm water management, reduce noise and air pollution, and favor habitat and biodiversity conservation, offer spaces for recreation, and enhance human health and well-being. Also, green roofs provide additional social and environmental benefits, including the possibility to use or re-use recycled materials in their construction and to produce bioelectricity exploiting living plants and microbial fuel cells (Adilkhanova et al. 2024; Tabatabaee et al. 2021; Francis and Jensen 2017).

With reference to the aspects that can be criticized about green roofs, such as the cost of maintenance, or the lack of monitoring of their impacts, perhaps the most notable is the controversy on whether green roofs are a source or sink of pollution in the urban environment (Vijayaraghavan et al. 2018, Marín et al. 2023). Regarding the used references, mainly a green roof can be considered a source of pollutants if the concentrations of these in its leachate are higher than the concentrations in the water before passing through the green roof. Therefore, the resulting effect of green roof leachates between source or sink is often based on two types of situations. The first one is the comparison of the runoff quality from green roofs with conventional roofs; the second is to compare leachate from green roofs with the quality of rainwater on the same surface during the same period of time. Some authors have highlighted the source effect of nutrients from green roofs. This effect contributes to the increase of nutrients in water bodies, specifically phosphorus (P) and nitrogen (N), which leads to water bodies’ eutrophication. Source effect results, in turn, in unwanted disturbances in the trophic chain balance in the water and affects, furthermore, the quality of the water itself (Li and Babcock 2014). It was found that the main causes of the deterioration of the quality of runoff water from green roofs are related, on the one hand, to the materials of the green roof, especially the substrate and drainage materials, and on the other hand, to fertilization activities during the service life of the roof (Czemiel 2010; Hachoumi et al. 2021; Volder and Dvorak 2014).

These are usually the most common causes in the case of P. Regarding this chemical element, some authors reported measurements of P in leachate from green roofs that exceed 1–2 mg/L, which is the maximum concentration allowed by international regulations on water quality (EU 91/271/ECC), EPA-US (EPA-US, 2023), and NPDES (NPDES, 2023). For example, total phosphorous concentrations were higher in leachate from green roofs than from conventional roof (Kok et al. 2016). Other studies compared the concentrations of P and phosphates present in green roof leachate to concentrations in rainwater (Monteiro et al. 2017; Karczmarczyk et al. 2018). There are also numerous case studies on nutrient determination, without the use of fertilizers in green roofs, where reductions of 60–80% in the concentration of P in leachate from green roofs were obtained (Marín et al. 2023).

In the case of N, a greater controversy regarding whether green roofs are a source or sink was reflected in the conclusions of the study by Castro et al. 2020 (Castro et al. 2020). In some previous studies, concentrations of N in leachate from green roofs were higher, source effect, than the benchmark value for control organisms (10 mg/L) (Shafique et al. 2018). This fact generates eutrophication in water bodies, among other environmental consequences, such as an increased acidity and toxicity of aquatic ecosystems, affecting the survival, growth, and reproductive capacity of some animals (Johnson et al. 2016). In terms of the source or sink effect of green roofs in the case of N, the results are slightly less conclusive than in the case of P. An example of these two scenarios was the research in which they concluded on the possible sink effect of green roofs for NO_3_ (Carpenter et al. 2016), compared with a source effect of green roofs for TN and NO_3_ due to higher concentrations compared to concentrations in rainwater (Okita et al. 2018).

Another important topic discussed in previous scientific studies is the content of metals in leachates from green roofs, as well as their chemical nature and environmental impact. This topic has been a subject of study in both a general way and specifically for certain metals (e.g., aluminum, zinc, or copper) in the processes of retention, detention, and infiltration within green roofs. Most of these studies mainly focus on metallic elements, and also on the metals, present in green roof construction materials. In studies that analyze the metals in leachate from green roofs, some authors use concentrations in the leachate from conventional soils or rainwater as benchmark figures (Gregoire and Clausen 2011). In general, it can be ascertained that international regulations on water quality are not usually referenced (Marín et al. 2023), and that represents the high added value of the present study. For example, very appropriately green roofs have been considered a source of heavy metals such as Ni, Sr, Mn, and Zn, without being considered dangerous in the bodies of water because the concentrations of these metals present in the leachates were below the limits of the regulations (Gregoire and Clausen 2011).

Therefore, although there is still some controversy about whether roofs are a sink or source of pollutants, it could be generally stated that they are usually a source of nutrients, and that the main cause is the substrate and subsequent maintenance fertilization (Wang et al. 2017a, b). On the other hand, and despite the different results found for metals, there is a general positive consensus that the substrate absorbed the pollutants and heavy metals and enhances the water quality (Mendes et al. 2024). In this context, it is necessary to seek solutions in order to reduce the release of nutrients into the environment, without compromising the normal development of vegetation and the proper functioning of green roofs, and maintaining equal or better performance in terms of metal remediation.

Recently, innovative green roof systems based on rock mineral wool (RMW) needled felt have been developed. These RMW-based green roof systems allow improving the capacity of the roof to retain runoff water (quantity) as well as the building thermal performance (Kostadinovic et al. 2022; Arkar et al. 2019). Furthermore, since these two improvements mainly impact the use phase of the system, in previous life cycle analysis research (cradle-to-grave perspective) it was concluded that, due to these two benefits, green roof systems using RMW as the substrate layer are comparable, in terms of environmental impact, to those GRs that use a conventional substrate (Vacek et al. 2017).

Since these green roof systems replace the conventional substrate with the RMW layer, nutrient leaching is no longer a problem. However, three aspects must be taken into account: (1) These extensive green roof systems are usually finished with a sedum blanket as a main layer of vegetation. Sedum blankets are supplied with a thin conventional substrate layer (1–2 cm), which could be a source of leachate. (2) In hot, dry climates, an extra layer of conventional substrate may be added to give the system a little more moisture inertia in order to ensure the plants’ survival. (3) On the other hand, the ability of these new green roof configurations using rock wool as a substrate to act as a sink or source of metals once integrated in the green roof profile is also unknown. Therefore, it is necessary to increase knowledge about the behavior of RMW-based green roof systems in terms of the quality of leachates in runoff water.

A particularly important issue that has been observed when reviewing the topic of water quality in runoff from green roofs is “what reference is used” to determine whether a system is a sink or source of nutrients or metals. In previous research, conventional soil leachates or rainwater have often been used as points of comparison (Castro et al. 2020) but only few studies make comparisons to the benchmark values established by official international control bodies, such as the WHO and EPA (Carpenter et al. 2016) for wastewater discharges. In the presented research, the limit values of current regulations on water quality have been considered to discuss from the obtained results whether a green roof system is a sink or source of some element. This procedure should be the standard in studies on leachates for green roofs, in order to avoid misinterpretations (Marín et al. 2023).

Thus, in order to investigate whether RMW-based green roof systems with needled felt can improve the quality of runoff water, this research aims on comparing the leachates from four different RMW-based green roof configurations, using 2- or 4-cm RMW blanket, and with and without an extra layer of substrate. The tested green roofs were compared to two reference systems: a conventional extensive green roof using pozzolana as a drainage layer, widely used in Mediterranean area, and a conventional flat roof ballasted with gravel. The results obtained have been analyzed using the limits of current regulations for wastewater discharge to determine whether they are sinks or sources of nutrients or metals.

## Material and methods

### Location and climate

This study was conducted at the University of Lleida, Spain, specifically located in the facilities of the Dr. Pius Quer i Font Arboretum, under dry continental Mediterranean climate. This climate corresponds to Csa: warm-temperate (C), summer dry (s), hot summer (a), according to the Köppen classification (Peel et al. 2007), characterized by cold and foggy winters while summers are hot and dry. The mean annual temperature is 15 °C, oscillating between 5.5 and 25.2 °C, with daily thermal amplitudes of 17–20 °C. The total rain average is 341.6 mm, distributed in 46.3 days with rain (≥ 1 mm) (reference period 1982–2016).

### Experimental set-up

**Fig. 2:**
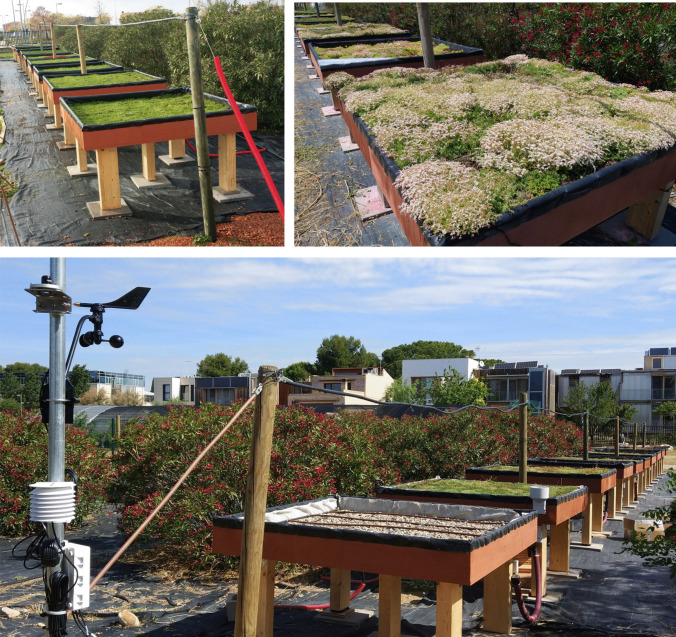
Experimental set-up at implementation (top left and bottom) and 2 years later (top right)

The experimental set-up consisted of six 1.8 × 1.8 m (3.24 m^2^) tables, sloped at 2%, simulating the six green roof systems to be compared (Fig. 2). These tables were designed to easily collect samples of runoff water, through an orifice with a tubular outlet in the lower part of each one of them.

The extensive green roof systems used are as follows.

- Extensive green roof with 2 cm of three-dimensional drainage and 2 cm RMW (sample 2)
- Extensive green roof with 2 cm of three-dimensional drainage and 2 cm RMW plus 5 cm of substrate (sample 2S)
- Extensive green roof with 2 cm of three-dimensional drainage and 4 cm RMW (sample 4)
- Extensive green roof with 2 cm of three-dimensional drainage and 4 cm RMW plus 5 cm of substrate (sample 4S)
- Extensive green roof with 4 cm of pozzolana as drainage plus 5 cm of substrate (sample PS)
- Conventional ballasted gravel roof (sample G)

**Fig. 3:**
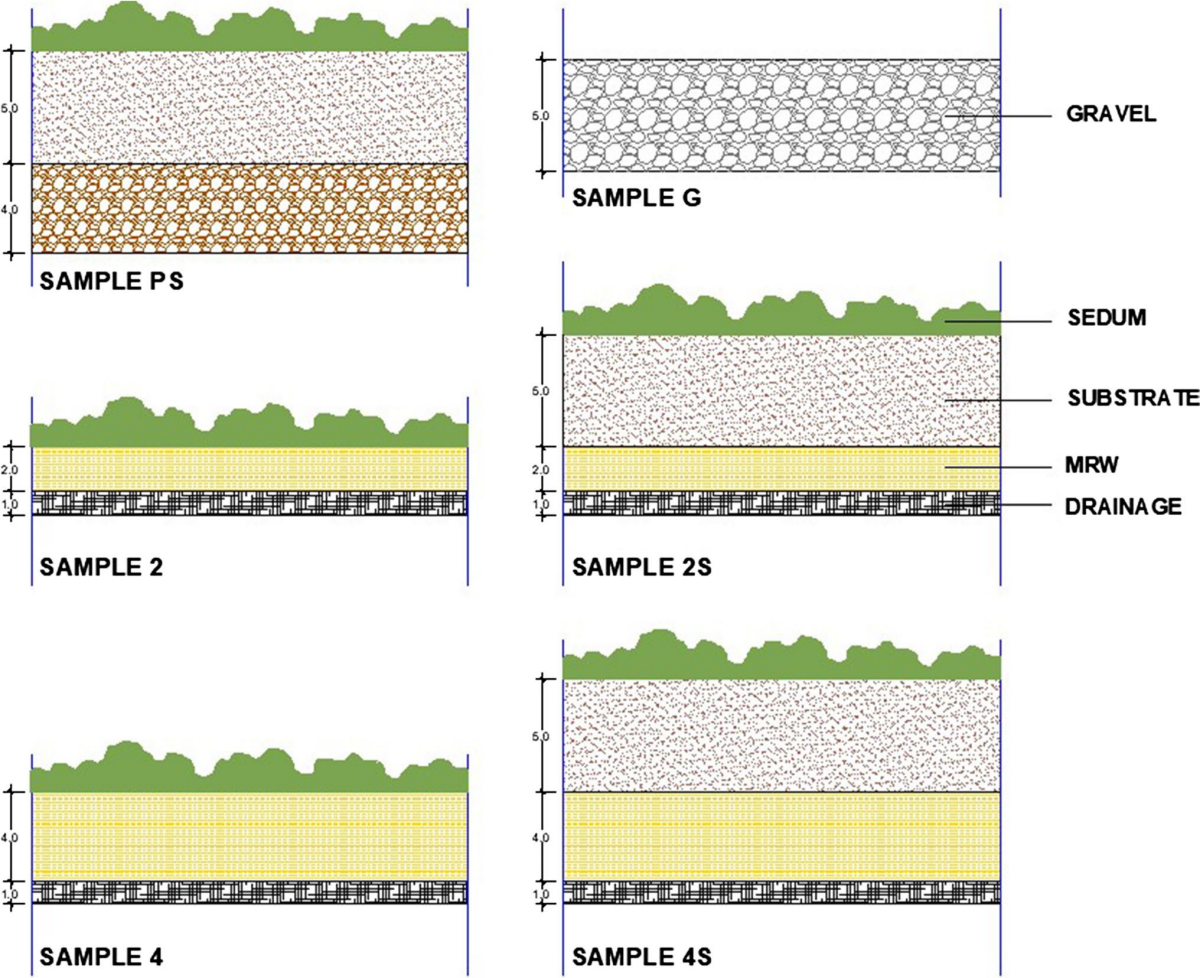
Sample profile description: PS, G, 2, 2S, 4, 4S (thickness in cm)

Table 1 and Fig. 3 show the main physical characteristics of the six systems analyzed. Systems with RMW needle felt are variations of the same substrate-less solution based on rock mineral wool layers (Fig. 3). The RMW-based green roof system consists of four main layers: (1) the root-proof membrane; (2) the drainage system, which can be with or without a water buffer; (3) the rock mineral wool needled felt, which acts as a growing medium and water retention layer; and (4) the vegetation layer, usually a sedum blanket (Urbanscape.com 2024). For this experiment, substrate-less solutions with RMW needled felt layers (samples 2 and 4) were compared with their equivalents but adding an extra 5-cm light-weight green roof substrate layer (samples 2S and 4S), according to the standard thickness substrate between 5 and 10 cm for extensive green roofs (Zhang et al. 2022).

**Table 1:** Main characteristics of the studied green roof systems

No	Sample name	Green roof system	Drainage layer (cm)	Root barrier layer (cm)	Rock mineral wool layer (cm)	Extra conventional substrate layer (cm)	Vegetation layer
1	2	RMW	2	0.02	2	-	Sedum blanket
2	2S	RMW plus substrate	2	0.02	2	5	Sedum blanket
3	4	RMW	2	0.02	4	-	Sedum blanket
4	4S	RMW plus substrate	2	0.02	4	5	Sedum blanket
5	PS	Pozzolana plus substrate	4	0.02	-	5	Sedum blanket
6	G	Gravel	7	0.02	-	-	-

The description of each of the layers is detailed below (Fig. 3):

- In this study, a drainage layer without a water buffer was used in all six tables. This layer is a three-dimensional, light and flexible composite matting made up of a drainage core of looped polypropylene filaments, which gives it a high drainage capacity, provided on both sides with a nonwoven filter fabric.
- Two different thickness, 2 and 4 cm, were used for the RMW layer, these being the ones used by the system supplier for green roof solutions (Urbanscape.com 2024).
- The “extra” substrate used in samples 2S, 4S, and PS was a commercial solution (Burespro.com 2024), especially designed for extensive green roofs, with a composition of 80% volcanic sand and 20% vegetal organic matter. Furthermore, the substrate included a base fertilizer (NPK + MgO) to facilitate root fixing and support initial plant growth.
- The vegetation layer consisted of a pre-grown *Sedum* sp. blanket with a mixture of *Sedum album*, *Sedum acre*, *Sedum hispanicum*, *Sedum spurium* ‘Summer Glory’, *Sedum sediforme*, and *Sedum sexangulare*. Among the available ones, *Sedum* species are commonly used in extensive green roofs due to their shallow root system, tolerance to climate extreme conditions, and its widespread use throughout the world (Pérez et al. 2020).

Two tables were used as benchmarks. First, a common extensive green roof system made of traditional materials, i.e., pozzolana as a drainage layer and substrate (sample PS), and second, a traditional flat roof ballasted with 7 to 9 cm of gravel (sample G). The gravel system is the most commonly applied solution in the building sector for flat roofs (Shafique et al. 2018), and the pozzolana-based roof is a widely used green roof solution (Pérez et al. 2015).

To study the leachates from the tables, samples collected periodically over 2 years were analyzed; leachates were collected every 3 months. A periodic leachate analysis requires enough sample, that is, a minimum amount of 1 L of runoff from each table at the same time, to be able to make the comparison in the same conditions for all the tables. Of this collected liter by table, 200 cm^3^ was finally used to carry out the physicochemical analysis. This process will depend on natural rainfall events, with the consequent uncertainty about its occurrence, or artificially forcing the runoff effect. According to previous studies (Li and Babcock 2014; Gregoire and Clausen 2011), only > 5 mm rainfall events were used to collect runoff samples for leachate analysis (Buffam et al. 2016). Given that rainfall in the study area is scarce and scattered, a periodicity of natural rain events to gather runoff samples could not be guaranteed. In Table 2, it can be observed that, in the study area, only 17.8 rain events of more than 5 mm and 9.5 of more than 10 mm take place on average. In total, there are no more than 15 rainy days per year. Thus, since there would not be enough water from natural rain to obtain the leachates, it was decided to force runoff from the tables, in order to be able to periodically collect leachates. Therefore, an artificial rain facility was installed to simulate rain events, allowing runoff samples to be collected every 3 months. In addition, this rain simulation system made it possible to sporadically irrigate during the summer periods for the plants’ maintenance and survival.

**Table 2:** Historic data on rainfall in Lleida from 2007 to 2017

Year	Days with rain > 5 mm	Days with rain > 10 mm	Rain events No consecutive days	Total annual rain (mm)
2007	15	6	14	226.5
2008	23	11	19	414.2
2009	21	13	19	443.6
2010	17	6	16	256.0
2011	16	11	16	287.3
2012	23	14	18	378.0
2013	20	14	14	382.6
2014	15	8	14	426.5
2015	12	6	12	265.2
2016	17	8	12	333.8
2017	17	8	13	341.6
**Avg**	**17.8**	**9.5**	**15.2**	**341.4**

The rain simulation system consisted in a set of Netafim SuperNet sprinklers (TuRiego 2024) with a flow rate from 40 to 110 L/h (at different pressure levels). They were located in the center of each table, at a height of 1 m above the vegetation layer, to homogenize irrigation and make the radius cover the entire table surface. This system made it possible to create artificial rainfall events, thus guaranteeing the generation of runoff to obtain the samples. In order to maintain the criterion between collection days, it was determined to take the samples at the beginning of the runoff effect from each table. With the aim to observe how the chemical elements already present in the system were released from implantation onwards, no fertilizer application, or any other maintenance activity that could potentially add matter to the system, was performed during the 2-year period.

### Data collection and analyzed parameters

In order to observe the evolution of runoff water quality throughout the 2 years of study, leachates were collected every 3 months. A total of seven suitable sample collections were obtained and reported.

The dates of sample collection were as follows: (1) first flush on 12.12.2018, (2) 20.06.2019, (3) 19.09.2019, (4) 09.12.2019, (5) 26.05.2020, (6) 08.09.2020, (7) 11.12.2020.

On 12.03.2019, it was registered an invalid collection due to a mixture in the water entering the system, between current irrigation water and water from a nearby irrigation channel.

The water samples were gathered in duplicate, for each tested green roof system, using a clean plastic laboratory flask, and directly transported to the laboratory, where they were stored in the refrigerator until being analyzed.

For the selection of the variables to be considered in the leachate analysis, on the one hand, previous research was taken into account (Czemiel 2010; Morales et al. 2017; Li and Babcock 2014; Gregoire and Clausen 2011). From these studies, some variables stand out, among others: dissolved solids, pH, conductivity, nutrients (especially N and P), and some metals. In particular, this selection of chemical parameters aims to establish and clarify the usual concentrations of the main chemical parameters influencing water quality present in leachates from green roofs, to finally draw conclusions about the impact of green roofs on urban runoff quality. Table 3 summarizes the analyzed parameters as well as the method used to measure them.

**Table 3:** Parameters analyzed from sample leachates

Parameter	Units	Description of methods used
Total suspended solids (TSS)	mg/L	Filtered with 1.2-µm pores and dried at 105 °C
Nitrate	mg NO3^−^/L	Nitrachek test
Electric conductivity (EC)	µS/cm	Potentiometry
pH		Potentiometry
Total organic carbon (TOC)	ppm	Combustion
Total inorganic carbon (TIC)	ppm	Combustion
Total carbon (TC)	ppm	Combustion
Total nitrogen (TN)	ppm	Combustion
P, Na, Ca, Mg, K, Si, Al, Fe, Ti, B, Zn, Zr, Cu	ppm/ppb	ICP-MS

### International water regulations considered

As mentioned above, the reference used in studies to determine whether a green roof is a sink or source of elements is crucial when interpreting research results. There are large variations in the runoff water quality and water retention by the different typologies of green roofs. Factors like rainfall characteristics, green roof design, fertilization, and maintenance influence the hydrological performance and runoff water quality of green roofs. Usually, in many studies, a green roof is considered a source of contaminants if the pollutant concentrations in the runoff are higher than those in the rainwater. Occasionally, there are studies that compare with the limits established by the official regulations on wastewater quality (Marín et al. 2023). For example, levels of PO_4_ and TP levels in all of the green roof runoffs were below the environmental standards of class IV waters (0.3 mg/L) for the protection of surface freshwater from threats of eutrophication, as well as below the standards for urban irrigation (0.5 mg/L) in Australia (Wang et al. 2017a, b). Also, some studies concluded that after comparing the quality of the runoff from the green roofs with wastewater regulations, the possible utilization for green roof runoff could be agriculture/forestry consumptions and urban non-potable purposes (Teemusk and Mander 2011; Liu et al. 2020).

In the present study, with the purpose of contextualizing the results in reference to current regulations on water quality, the values for each analyzed parameter were compared with the limits established in current international regulations from Europe, the USA, and Asia. Thus, considering that the utilization of runoff from green roofs for urban non-potable purposes is possible, we have considered the following wastewater regulations:

- European Union
  **EU** COUNCIL DIRECTIVE of 21 May 1991 concerning urban wastewater treatment (EU 91/271/EEC).
- USA: Environmental Protection Agency (EPA-US, 2023)
  National Pollutant Discharge Elimination System (NPDES, 2023).
- Asia: Water Environment Partnership in Asia (WEPA).
  WEPA is an initiative of the Ministry of the Environment of Japan. It is registered as a project aimed at water pollution prevention and ecosystem, including “domestic wastewater treatment” and “climate change and the water environment” (WEPA, 2023).
- **Environmental Protection Agency (EPA)** of Ireland, 2001, provides some “Parameters of Water Quality (Interpretation and Standards)” which prove very useful for assessing the limit values of the various parameters and chemicals that determine the quality of wastewater.
  Table 4 summarizes the values from these wastewater regulations for each physicochemical parameter considered in Table 3.

**Table 4:** Values considered for wastewater of each regulation

Parameters	Units	Wastewater regulation			
		EU 91	EPA Ireland	EPA USA	WEPA
TSS	mg/L	35–60	50	62	30–60
pH		6.5–9.5	5.5–9.0	6.5–8.5	-
EC	µS/cm	-	1000	-	-
TN	mg/L	10–15	-	-	30–40
NO_3_	mg/L	-	50	-	-
TP	mg/L	1.0–2.0	-	1	-
Na	mg/L	-	200		200–250
Al	ppb	-	200	50–200	100–200

## Results and discussion

### First flush characterization

The first leachate collection event was on 12 December, 2018, and the results from this collection are considered the “first flush.” Previous studies asserted that first flush events have a high influence on the quality of runoff water leaving green roof systems (Czemiel 2010; Li and Babcock 2014; Gregoire and Clausen 2011), specifically when the system includes substrate and therefore the presence of nutrients like P and N (Buffam et al. 2016). Table 5 summarizes the parameters and their values after the first flush event for all six samples.

**Table 5:** Summary of values obtained for all the studied parameters in the first flush event

First flush analysis						
		Average RMW systems	Average RMW with substrate systems	Pozzolana with substrate system	Ballasted gravel system	Irrigation water
TSS	mg/L	22.00	15.00	52.0	329.0	5.0
pH		7.45	7.75	7.9	7.7	8.0
EC	µS/cm	378.15	757.25	453.2	392.5	357.0
TOC	ppm	5.25	21.00	12.3	10.0	6.3
TIC	ppm	13.80	20.35	24.9	25.2	24.7
TC	ppm	19.10	41.35	37.2	35.2	31.0
TN	mg/L	0.70	28.35	8.0	3.9	2.1
NO_3_^−^	mg/L	5.00	112.50	8.0	41.0	5.0
P	mg/L	3.65	11.15	10.1	3.9	1.6
Na	mg/L	100.95	303.15	208.0	149.7	98.8
Ca	ppm	106.65	301.10	588.1	854.2	80.5
Mg	ppm	57.60	183.75	169.0	110.7	46.5
K	ppm	65.90	130.25	109.8	46.6	14.7
Si	ppm	52.15	82.35	175.1	278.9	29.3
Al	ppb	7.50	6.50	10,466.0	24,579.0	11.0
Fe	ppb	45.00	38.00	3855.0	21,411.0	8.0
Ti	ppm	0.00	0.05	3.6	2.5	0.1
Cr	ppb	0.50	0.50	2.0	32.0	2.0

To better understand the results from the phytochemical parameters analyzed in Table 5, below they are grouped according to the following topics: nutrients, metals, pH, TSS/EC, and carbon.

#### Nutrients

All the four studied systems as well as the irrigation water exceed the limit of the wastewater regulations for TP. Among the diversity of P compounds, phosphate ion and elemental P are the assimilable forms in the water. The P present in the water is the measure of the phosphates in the water and the measure of elemental P. Thus, the measurements of phosphates and elemental P can be considered together (summed), like the measurement of total phosphorus (TP). The RMW system (without substrate) had the lowest concentration of TP with 3.65 mg/L. The two systems with substrate had the highest concentration of TP. Commonly, the substrate increases the P content in green roof runoff (Teemusk and Mander 2007; Toland et al. 2012). Studies relating to substrate and depth evaluations concluded that in leachate from green roofs the concentration of TP was higher compared to conventional roofs, and at 15-cm depth of substrate than at 5-cm depth (Liu et al. 2020; Gong et al. 2019). Also, the leachate from unfertilized green roof had a TP concentration of 0.14 mg/L, while the leachate from fertilized green roof had TP concentrations between 1.57 and 1.82 mg/L (Zhang et al. 2015; Bus et al. 2016). In general, leachates from green roofs contain amounts of P, mainly coming from the substrate and from the fertilizers used to maintain the green roof.

The RMW system without substrate acted as a sink for TN and NO_3_ with values of 0.70 and 5.0 mg/L, respectively. Comparing the concentrations of TN and NO_3_ from the irrigation water and the limits of the regulation, the concentrations of TN and NO_3_ from the runoff of the RMW system were below. The systems with substrate had the highest values for TN and NO_3_. The chemical complexity of N is given by its seven different oxidation states (from 3 to + 5). The most common ionic (reactive) forms of inorganic N in aquatic ecosystems are ammonium and non-ionized ammonia, nitrite, and nitrate. Thus, the measurements of ammonium and non-ionized ammonia, nitrite, and nitrate can be considered together (summed), like the measurement of TN. Various studies have found the capacity of green roofs to retain N, that is, a sink effect (Chen et al. 2018; Van Seters et al. 2009; Mitchell et al. 2018). The studies that confirm the source and/or sink effect of green roofs for the different forms of N are divided by the influence of the fertilizers. Several researchers have found that concentrations of different forms of N are lower in runoff from green roofs (0.156 mg/L—NO-3) than in rainwater and conventional soil (0.412 mg/ L—NO-3) (Shafique et al. 2018; Van Seters et al. 2009), while others have indicated that there are significant concentrations of N in runoff from green roofs (Beecham and Razzaghmanesh 2015). In the present study, fertilizers were not applied, with the aim of knowing the leachates coming from the systems studied.

#### Metals

There is a divided result between sink or source effect for the systems with and without substrate and compared with the irrigation water, according to the concentrations obtained for metals. Metals like Cr, Ti, and Al resulted in a sink effect in RMW system compared with RMW with substrate system. Comparing the RMW systems with and without substrate, Fe, Si, K, Mg, and Ca had lowest concentration in RMW systems with substrate. A sink effect resulted for Cr, Ti, and Al in systems RMW compared with the irrigation water and the limits of the regulation for wastewater. Among the components of green roofs, the substrate layer is essential for the retention of heavy metals, being able to achieve a removal percentage in green roof leachates of up to 97% (Vijayaraghavan and Raja 2014). Results showed that the proportions of heavy metals from the green roof runoff depend mostly on the substrate type and the fertilizer used (Malcolm et al. 2014).

#### pH

There are numerous studies that affirm the ability of green roofs to mitigate acid rain problems, increasing pH from 5.0 to 6.0 and from 7.0 to 8.0 (Qianqian et al. 2019; Wang et al. 2017b). All the tested systems acted a sink effect comparing with the irrigation water and the limits of the regulations. All the leachates from the studied green roofs systems had lowest pH than the irrigation water used during the experiments.

#### TSS and EC

According to previous studies (Pęczkowski et al. 2020; Akther et al. 2020; Speak et al. 2014), due to the contributions of organic content mainly from the substrate, the green roof leachates presented higher levels of both TSS and electrical EC than the irrigation water. In our study, it was observed that the amount of TSS leached from conventional gravel-ballasted roofs is much greater than from green roofs. The EC was higher on green roofs, although it is clearly related to the substrate, since RMW systems without substrate are the ones that have the lowest EC, with a value close to that of the inlet water.

#### Carbon

TOC is used to measure the organic molecule content in water (Zhang et al. 2015). The RMW system without substrate resulted in the best average for TOC, TIC, and TC compared with the rest of the systems and with the irrigation water. The possible elevated TOC concentrations in runoff of green roof originated from the organic matter in the soil and the decaying vegetation. Studies confirm that certain materials like asphalt fiberglass are likely to be sources of TOC, and then TOC concentrations in asphalt roads are 3–5 times higher than those in runoff from a concrete road (Mendez et al. 2011).

Finally, after discussing the first flush effect, and at view of the results from Table 5, we concluded that the RMW-based green roof systems without extra layers of substrate performed better than those with extra substrate layers. The results for RMW green roof systems with no extra layer of substrate suggest that the design of green roofs with a layer of RMW could be a good option for reducing TOC leachate. For all the phytochemicals parameters presented in Table 5, except for TSS, K, and Fe, RMW systems without substrate had the lowest concentrations compared with the rest of the tested systems.

### Runoff water quality during the 2-year monitoring period

**Fig. 4:**
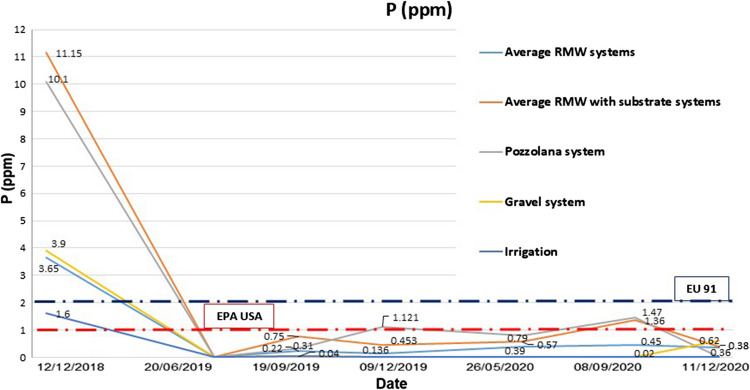
Evolution of phosphorous leachates from all samples during the 2-year study. Average values compared to international regulations

After the first flush, the values of the concentrations for the different phytochemicals parameters became stable, with slight variations from one sample to another through the time. Previous studies reached similar conclusions, stating that, over time, the green roof was effective in reducing storm water runoff and reducing the concentrations of most pollutants in leachates (Gregoire and Clausen 2011; Teemusk and Mander 2011). As an example, Fig. 4 shows the evolution of P over the course of the experiment’s 2 years. The *first flush effect* is identified perfectly during the first simulated rainfall with high and far values of the rest simulated rainfalls (differences from 12 December 2018 to 11 December 2020). From 20 June 2019, the release of leachates stabilized, in this case for P with concentrations between 1.0 and 2.0 mg/L.

The mean values obtained during the whole experiment (2018–2020), without considering the first flush, provide very interesting data since they can be contrasted with the limits of international water quality regulations. Table 6 shows the average values without considering the first flush and differentiating between systems with no addition of lightweight green roof substrate and systems which included an extra substrate layer, as well as the reference systems (pozzolana with substrate and conventional roof ballasted with gravel). The last column in Table 6 shows the quality parameters of the water inlet used as a base for the comparative study.

**Table 6:**
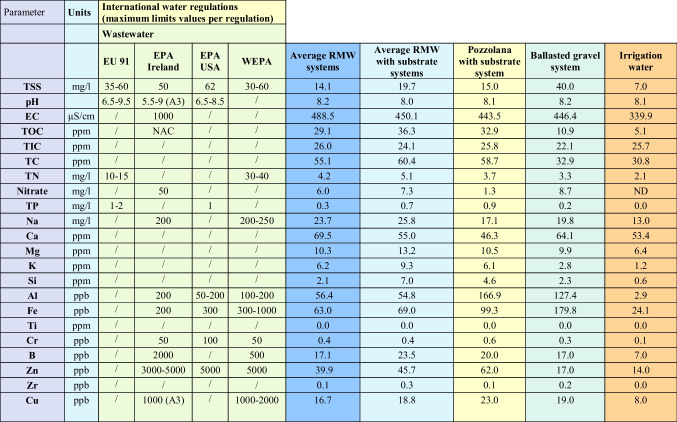
Summary of the maximum limit values for wastewater regulations and the average values obtained for all the studied parameters

From Table 6, it can generally be inferred that the RMW system without substrate had the best behavior compared with the rest of the tested systems with substrate (RMW and pozzolana).

Also, the RMW systems with and without substrate had better behavior for the retention of metals like Al, Fe, Cu, Cr, and Zn compared with pozzolana with substrate system and inclusive with ballasted gravel system. It is important to highlight the concentration of TP in the runoff for both RMW systems, which were below from the pozzolana with substrate system and below of the limits for wastewater regulation.

Finally, it is noticeable that all tested green roof systems show a very good performance in terms of quality of runoff since for each single analyzed parameter the obtained values were below the limits in wastewater regulations (Table 6). In this context, RMW systems without extra layers of substrate provided very good results in retention of metals and nutrients.

In the following sections, the main results derived from the values in Table 6 are described in detail and discussed.

#### Total suspended solids (TSS)

**Fig. 5:**
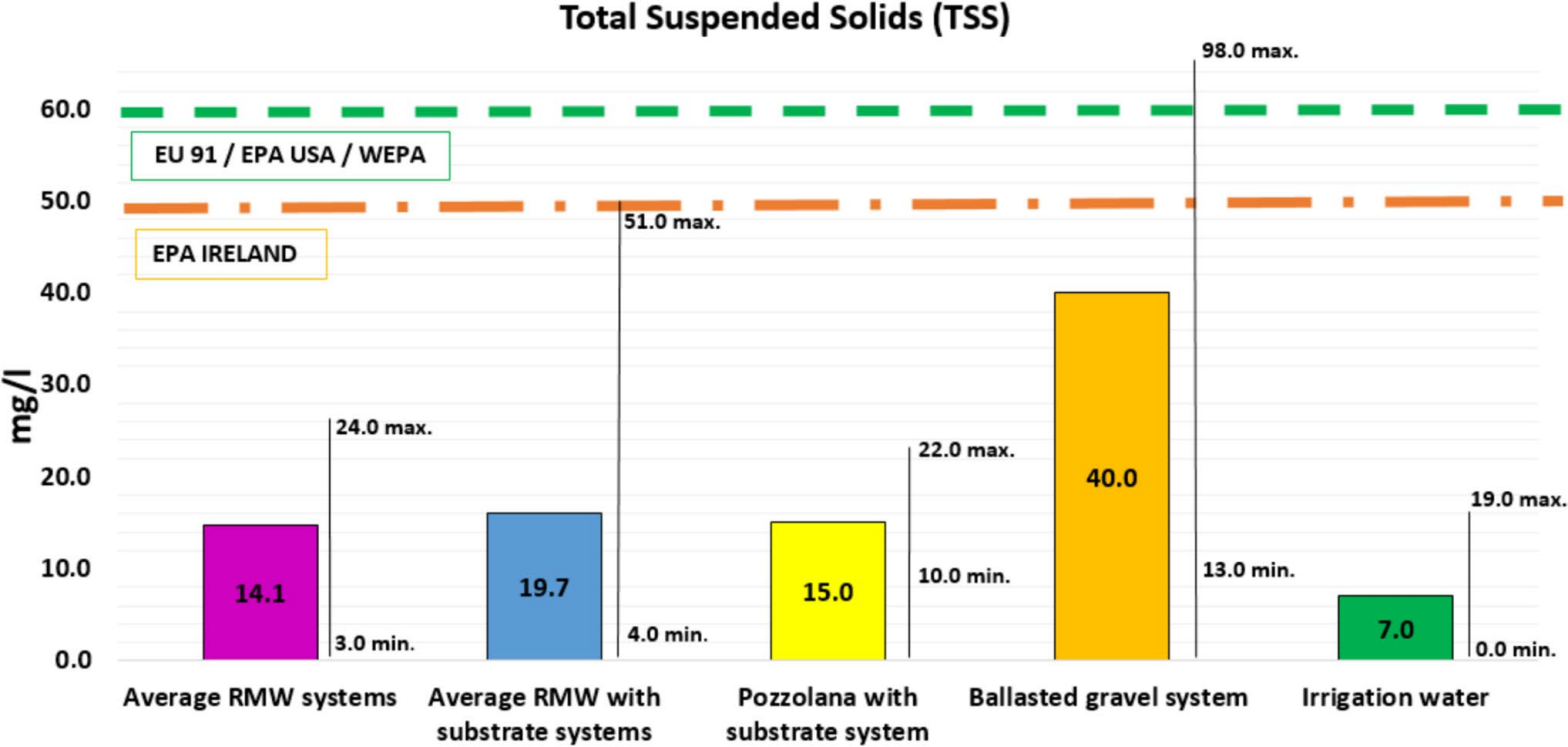
Total suspended solids (TSS). Average values compared to international regulations, and maximum and minimum values

Figure 5 shows the detailed results for total suspended solids (TSS). As expected, and in agreement with previous studies (Pęczkowski et al. 2020; Akther et al. 2020; Speak et al. 2014), the green roof leachates presented higher levels of both TSS and electrical conductivity (EC) than did the water taken as a benchmark, whether rainwater or irrigation. This is due to the contributions of particles mainly from the substrate.

According to the results of this experiment, the RMW systems have registered levels of TSS leachates below those established in the international standards for urban runoff water, ranging from 3 to 24 mg/L TSS with an average of all the tested systems of 14.1 mg/L TSS. Occasionally, slightly higher values of TSS have been recorded in some samples, a fact which can be explained by occasional accumulations of dirtiness in the runoff water outlets from the experimental tables. These figures clearly indicate the importance of green roofs in controlling TSS levels in comparison with non-vegetated roofs with ballasted gravel system.

#### pH

According to the literature, green roofs tend to neutralize acid rain and increase pH (Qianqian et al. 2019). Green roofs can mitigate acid rain damage by increasing pH (from 5.0–6.0 to 7.0–8.0) (Lim et al. 2021; Liu et al. 2020). The average pH of leachate from green roofs was 8.0 (Chen et al. 2018), while the pH values measured at the exit of the green roof were mostly higher than 7 (Gnecco et al. 2013).

In this experiment, a similar effect has been observed as pH was stabilized around a value of eight units (Table 6). It should be noticed that working with green roof RMW systems with needled felt does not appear to have had any consequences in pH throughout the experiment. Occasionally, a pH of 9 or higher has been reached in some of the samples. This occasional increase may be due to the decomposition of organic matter during the summer months. It should also be taken into account that the water in the area of study usually has a high pH value. Indeed, the water used for irrigation/simulated rains, that is, to generate runoff in leachate collection events, had an average pH during the 2-year study of 8.1.

#### EC and cations

As previously mentioned in the section on “Total suspended solids (TSS),” earlier studies have generally identified the substrate as the main cause of the higher TSS and EC values in leachate from green roofs. Previous studies concluded that EC and TSS contents are higher in green roofs than in precipitation and control roofs, due to the presence of these elements in the green roof substrate (Buffam et al. 2016). In the present experiment, similar values were measured in all systems. Although EC was slightly higher in leachates from RMW needled felt systems, the average value obtained was clearly below the limits established in international regulations (Table 6). Regarding the EC of the irrigation water, the measured values were consistent throughout the experiment, with an average of 339.9 μS/cm. Similar results were achieved in the study where in the analyzed runoff waters from the experimental surfaces, conductivity has higher values in relation to runoff from the control surface and rainwater (Pęczkowski et al. 2020). Although, due to the specific characteristics of the study area this value was higher, approximately double, than that reported in previous studies (Buffam et al. 2016; Pęczkowski et al. 2020; Buccola and Spolek 2011), it was within the internationally accepted values of EC for irrigation water in agriculture. Most surface irrigation water, whose source is snow-fed rivers, has a total salinity of less than about 500 to 600 μS/cm (Abrol et al. 1988).

Cation levels in leachates were slightly higher than in simulated rainwater. The water regulations reviewed only set maximum limits for sodium (Na) (Table 6), and the values obtained were far from these limits, so they are not noxious for the environment. Even in the case of calcium (Ca), the system with pozzolana and substrate drainage (PS) showed a lower average than those of the water inlet.

#### Carbon

TOC is used to measure the organic molecule content in water (Zhang et al. 2015), which comes from organic matter in the substrate and decomposing vegetation. The following measurements of TOC in wastewater can be taken as benchmark figures (Henze 2002): 250 mg/L (concentrated), 180 mg/L (moderate), 110 mg/L (diluted), and 70 mg/L (very diluted). In view of the results (Table 6), it can be concluded that measured TOC values have remained at the level of “very diluted” and occasionally “diluted” throughout the 2-year research period and for all tested systems (EU 98/83/EC).

Looking at the results for RMW green roof systems with needled felt, with no extra layer of substrate, it can be concluded that the thin layer of substrate (1–2 cm) that supports the sedum blanket also contributes to the release of leachate from the green roof.

Although most of the previous studies where TOC measurements are considered commonly highlight the big difference between the values for green roofs with and without substrate, especially when compared to rainwater (EPA/Ireland; Toland et al. 2012), in our case, the concentrations were not so large. This fact suggests that the design of green roofs with a layer of RMW could be a good option for reducing TOC leachate.

#### Nitrogen and phosphorous

Nitrogen and phosphorus leachate did not in any case exceed the limits set out in the guidelines for urban water discharges (Table 6). However, the contribution of the substrate to the discharge of nutrients (N and P), which is more noticeable in systems with an extra layer of substrate, can be observed in results of Table 6. As for TN, the RMW green roof systems with needled felt both with and without substrate, with values of 5.1 and 4.2 mg/L, respectively, presented higher mean values than did the benchmark roofs, with measurements of 3.7 mg/L for the green roof with pozzolana (PS) and 3.3 mg/L for the ballasted gravel roof (G). These quantities of N can be explained due to the joint contribution from the substrate and irrigation. Significantly, all systems fell below the maximum limit of 10 mg/L established in the regulations (EU-91).

Given that the 2.1 mg/L of N detected in the irrigation water (used as a benchmark) was higher than the values observed in previous studies (Qianqian et al. 2019), the N present in the water inlet could arguably be an important factor that directly influenced the final value in the TN leachates. In a similar study performed in the province of Lower Silesia, Poland, a TN value of 8.3 mg/L was obtained from a green roof while the benchmark TN value of the water inlet was 2.92 mg/L (Pęczkowski et al. 2020).

Regarding P, the RMW-based green roof systems registered lower average values, of 0.7 mg/L and 0.3 mg/L with and without substrates, respectively, than did the conventional green roof with pozzolana, with an average of 0.9 mg/L, but higher than the roof ballasted with gravel, which registered 0.2 mg/L. Taking into account that the regulatory limit is 1 mg/L (EPA-USA; EU-91), the solutions with RMW-based green roof systems with needled felt would seem to provide better performance than the conventional pozzolana in terms of P leachates. This result is very interesting given the environmental connotations due to eutrophication processes of natural water bodies as a consequence of the excess concentration of P as a nutrient in urban runoff water. When compared to the results obtained in previous studies, the results of the present study show similar but slightly higher results with regard to P leachates. In previous studies without the use of fertilizers, the results are more similar to the results obtained in the present study for solutions without substrate (0.3 mg/L). Thus, the concentrations of the green roofs studied were similar, with results of concentrations of TP of 0.24, 0.35, and 0.41 mg/L, which one of them had lower concentration of TP compared with the control of 0.27 mg/L of TP (Gong et al. 2019). While in others studies recorded lowest concentrations of TP of 0.11 and 0.14 mg/L in the leachate from green roofs (Zhang et al. 2015; Toland et al. 2012; Jennett and Zheng 2018). In contrast, in previous studies in which fertilizers were used, the results were similar to those recorded in our study for green roofs with an extra layer of substrate (0.6 mg/L). This is the case of the study in which TP concentrations ranging between 0.07 and 0.87 mg/L were obtained in the leachates from green roofs (Teemusk et al. 2011).

In green roofs with a roof slope of 15%, and using fertilizers for its maintenance, resulted with a TP concentration of 1.1 mg/L in leachates (Castro et al. 2020). This concentration is almost twice as high as the results of the present study, which has a slope of only 2% for all tested green roof solutions (flat roofs), and registered mean values of 0.6 mg/L in the leachate of TP. Finally, average concentrations between 0.1 and 0.17 mg/L of TP in the leachate of fertilized green roofs (Chen et al. 2018; Liu et al. 2020). These values were much lower than in the present study.

#### Metals

**Fig. 6:**
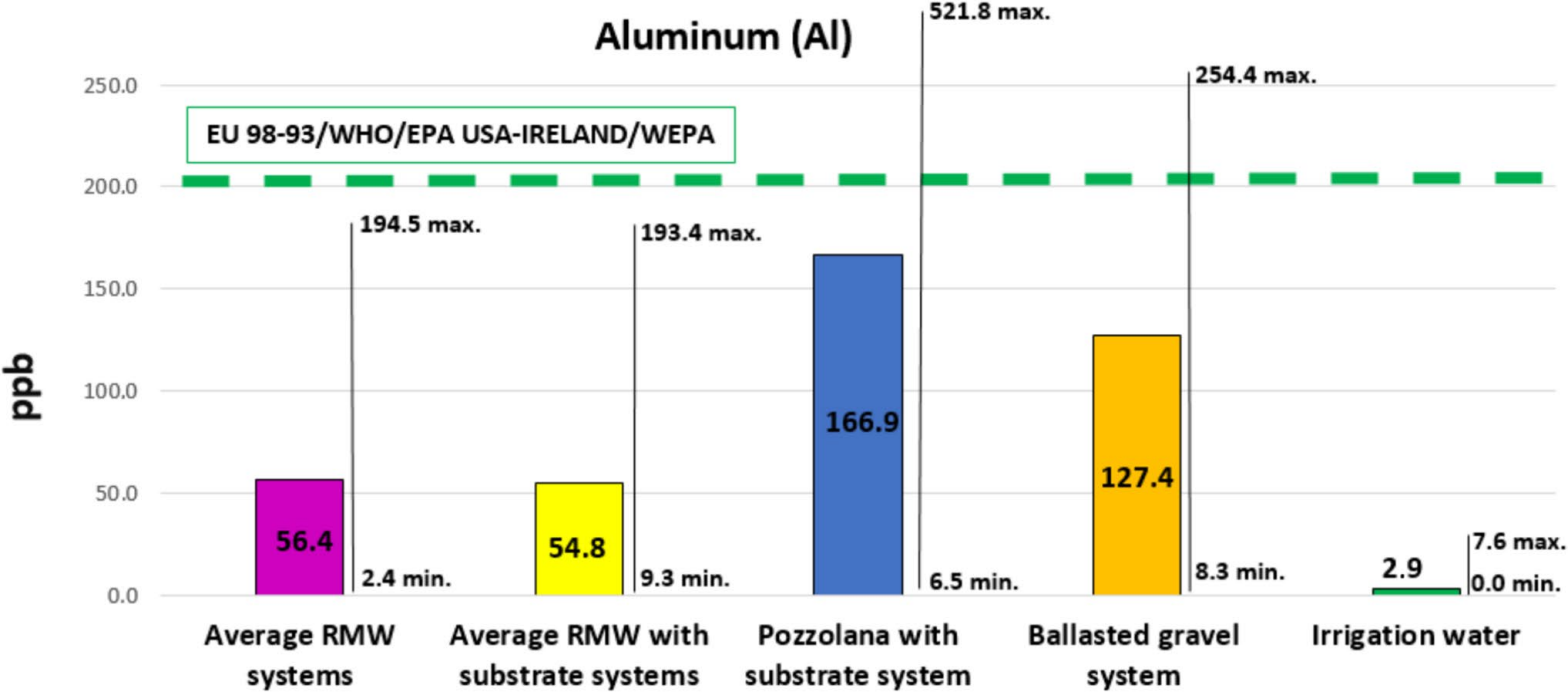
Aluminum. Average values compared to international regulations, and maximum and minimum values

**Fig. 7:**
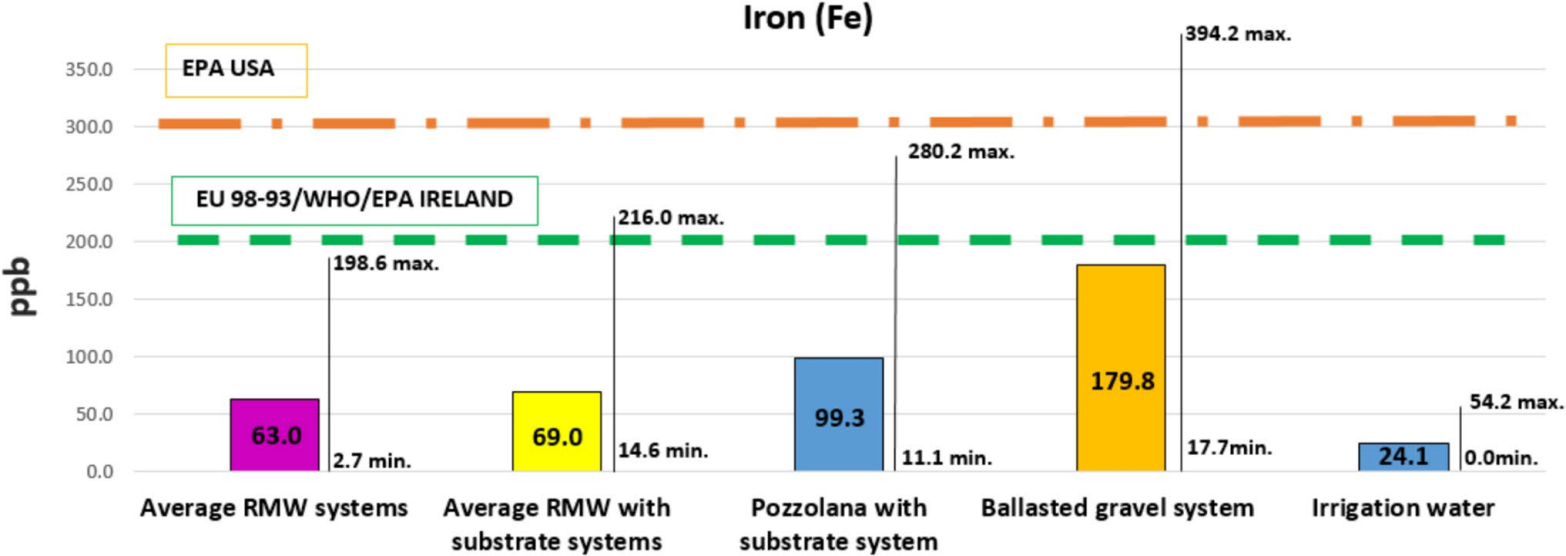
Iron. Average values compared to international regulations, and maximum and minimum values

As shown in Table 6, metal leachates from RMW green roof solutions generally fall within the limits set by urban water regulations. This fact is very positive, as metals are hazardous elements with the most negative impact on the environment. Figure 6 and Fig. 7 show the average results for aluminum and iron compared to current international regulations and the maximum and minimum values reached during the experiment.

It can also be observed that benchmark solutions, both the solution with volcanic gravel (pozzolana) and the conventional ballasted gravel roof (no green roof), leached more iron and aluminum than the solutions based on RMW, with averages closer to the limits of the regulations and maximum measured values higher than limits set in regulations. Iron leachates from green roof solutions with an extra layer of substrate also stand out, which confirms the source effect of the substrate layer. This fact highlights the advantages of using solutions based on RMW, without substrate. Nonetheless, it must be remembered that the substrate provides, in addition to physical support and nutrients for the plants, an extra water retention capacity that guarantees plant survival in climates with high solar radiation and low ambient humidity, e.g., the Mediterranean climate. Note that sedum blankets are supported by a thin layer of substrate that also contributes to leachates.

In previous studies, there is still controversy about the sink or source effect of metals from the substrates. Some authors highlighted that, among the different components of green roofs, the substrate layer is fundamental for the retention of metallic elements. Thus, according to these authors, a substrate layer is able to achieve, depending on its composition and thickness, a percentage of removal in green roof leachates up to 97% (Vijayaraghavan and Raja 2014). On the contrary, other emphasized that the substrate itself can be a source of metals (Wang et al. 2017a, b). In the present study, it was possible to observe that the iron and aluminum leachates are related to the mineral components of the green roofs, mainly the substrate and the pozzolana. Likewise, the gravel of conventional ballasted roofs is also a source of iron and aluminum. Therefore, decreasing the use of substrate and mineral materials and replacing them with more inert solutions such as RMW can be very beneficial for the environment in terms of reducing metal leachates. Most studies state that green roofs are optimal systems over time for the reduction of metallic elements in runoff water (Gregoire and Clausen 2011; Kuoppamäki and Lehvävirta 2016; Okita et al. 2018). In some studies, the concentrations of Fe in the runoff from the green roofs were lower compared to those of the conventional roof (Castro et al. 2020; Van Seters et al. 2009; Speak et al. 2014). On the other hand, metallic elements like Fe reported higher concentrations in leachate from green roofs when compared to the concentrations present in both rainwater and conventional soil runoff (Malcolm et al. 2014; Ferrans et al. 2018).

## Conclusions

In the presented research, four different RMW-based green roof configurations, using 2 or 4 cm RMW blanket, and with and without an extra layer of substrate were studied in terms of their capacity to improve the quality of urban water runoff. The tested green roofs were compared to two reference systems: a conventional extensive green roof using pozzolana as a drainage layer, widely used in Mediterranean area, and a conventional flat roof ballasted with gravel.

These substrate-less green roof systems are promising because they incorporate smaller amounts of substrate, avoiding some problems associated with nutrients leaching into the environment. The study considered the controversy over whether green roofs are sinks or sources of certain elements, often related to (a) the first flush effect, and (b) the reference used to interpret the results, whether specific control roofs, rainwater or irrigation water, etc.

For a long time, there has been controversy over whether green roofs are a source or sink of pollution in the urban environment. On the one hand, there are authors who defend the ability of the substrate to capture pollutants and of plants to fix pollutants in their growth. At the same time, other authors assert that the level of leachates is closely linked to the substrate layer and subsequent maintenance activities.

Few authors have considered whether or not the measured levels of the various chemical elements are within the current regulatory limits or if they really imply a problem for the environment compared to traditional roof systems. The quality of runoff water from green roofs can be affected by the leachates of N and P. The results of some studies indicate that P discharges generally exceed concentrations found in leachate from conventional roofs and/or rainwater. In the most occasions, the concentrations of P in leachate from green roofs exceed the 1 mg/L standard of the related regulations, while in most cases N, although being more leachable than P, is lower than the standard of 10 mg/L.

The results showed that solutions with RMW-based green roof systems with needled felt, especially those with no substrate, or “substrate-less” solutions, caused leachate levels below the limits of current regulations and also showed better performance than even traditional roofs, both the green roof with pozzolana and the traditional gravel-ballasted flat roof.

Since RMW blankets have a large capacity for water storage, the use of RMW-based green roof systems with needled felt with no substrate layer, or even using a thin substrate layer, could improve both the quantity and quality of urban runoff water.

Specific conclusions from this study are:

- **First flush analysis:**
  - The first flush effect took place in all six systems analyzed.
  - The TSS leachates from the pozzolana plus substrate (PS) and ballasted gravel (G) systems stood out over the other systems.
  - RMW-based green roof systems without extra substrate layer performed better than those with extra substrate layers, and better than pozzolana (PS) and gravel (G) systems as well, keeping leachate levels within the regulatory limits (except in the case of P).
  - The extra layer of substrate in most parameters was the cause of the large amount of leachates, in comparison to the leachates during the experiment after the first flush.
- **RMW-based green roof systems with needled felt during the 2-year monitoring:**
  - After the first flush, RMW-based green roof systems showed very good results, performing even better than pozzolana and ballasted gravel systems.
  - All the average values for the studied parameters (Table 6) of RMW-based green roof systems fell within the limits established in international regulations for urban water.
  - RMW-based systems showed lower leachates of most metallic elements (Al, Fe, Ti, Cr, B, Zn, Cu) than pozzolana and gravel systems.

In general, it can be stated that RMW green roof systems with needled felt, especially as a consequence of being substrate-less, improve the parameters relating to the quality of runoff water from green roofs. The comparison with gravel-ballasted roofs and pozzolana green roof systems showed clear advantage of RMW-based systems.

In this article, the benchmark values of the different water quality parameters established in the international regulations for wastewater have also been provided, which has made it possible to accurately describe the potential environmental impact associated with leachate from green roofs, concluding that it is minimal and may also be further reduced by solutions such as substrate-less RMW-based green roof systems.

Although the present study has certain limitations regarding the dimensions of the experimental tables, as well as the specific conditions of the experimental site, in reference to real roofs located in densely populated environments, the results are very valuable from the point of view of the contribution of green roofs, and especially those based on RMW, to the improvement of the quality of urban water runoff. In future research, it would be interesting to work on real cases and with longer research periods.

## Data Availability

All data produced in the study are provided in the tables and figures in the “Results and discussion” section.
